# Using hospital auxiliary worker and 24-h TB services as potential tools to overcome in-hospital TB delays: a quasi-experimental study

**DOI:** 10.1186/s12960-020-0457-2

**Published:** 2020-04-03

**Authors:** Miguelhete Lisboa, Inês Fronteira, Paul H. Mason, Maria do Rosário O. Martins

**Affiliations:** 1 Centro de Investigação Operacional da Beira (CIOB), Instituto Nacional de Saúde (INS), Rua Correia de Brito #1323 – Ponta-Gea, Beira, Mozambique; 2 Global Health and Tropical Medicine, Instituto de Higiene e Medicina Tropical (IHMT), Universidade NOVA de Lisboa (UNL), Rua da Junqueira No. 100 |, 1349-008 Lisbon, Portugal; 3 School of Social Sciences, Monash University, Wellington Road, Clayton, Victoria 3800 Australia

**Keywords:** Tuberculosis, Hospital auxiliary workers, 24-h TB laboratory using Xpert, Same-day TB diagnosis and treatment, Hospital TB mortality

## Abstract

**Background:**

In-hospital logistic management barriers (LMB) are considered to be important risk factors for delays in TB diagnosis and treatment initiation (TB-dt), which perpetuates TB transmission and the development of TB morbidity and mortality. We assessed the contribution of hospital auxiliary workers (HAWs) and 24-h TB laboratory services using Xpert (24h-Xpert) on the delays in TB-dt and TB mortality at Beira Central Hospital, Mozambique.

**Methods:**

A quasi-experimental design was used. Implementation strategy—HAWs and laboratory technicians were selected and trained, accordingly. Interventions—having trained HAW and TB laboratory technicians as expediters of TB LMB issues and assurer of 24h-Xpert, respectively. Implementation outcomes—time from hospital admission to sputum examination results, time from hospital admission to treatment initiation, proportion of same-day TB cases diagnosed, initiated TB treatment, and TB patient with unfavorable outcome after hospitalization (hospital TB mortality). A nonparametric test was used to test the differences between groups and adjusted OR (95% CI) were computed using multivariate logistic regression.

**Results:**

We recruited 522 TB patients. Median (IQR) age was 34 (16) years, and 52% were from intervention site, 58% males, 60% new case of TB, 12% MDR-TB, 72% TB/HIV co-infected, and 43% on HIV treatment at admission. In the intervention hospital, 93% of patients had same-day TB-dt in comparison with a median (IQR) time of 15 (2) days in the control hospital. TB mortality in the intervention hospital was lower than that in the control hospital (13% vs 49%). TB patients admitted to the intervention hospital were nine times more likely to obtain an early laboratory diagnosis of TB, six times more likely to reduce delays in TB treatment initiation, and eight times less likely to die, when compared to those who were admitted to the control hospital, adjusting for other factors.

**Conclusion:**

In-hospital delays in TB-dt and high TB mortality in Mozambique are common and probably due, in part, to LMB amenable to poor-quality TB care. Task shifting of TB logistic management services to HAWs and lower laboratory technicians, to ensure 24h-Xpert through “on-the-spot strategy,” may contribute to timely TB detection, proper treatment, and reduction of TB mortality.

## Contributions to the literature

- The use of layperson in hospital settings and routine availability of molecular TB diagnostic tools are known to be helpful in speeding up early TB diagnosis and treatment.
- Our study found that having HAWs as expediters of logistic and operational TB matters and 24-h availability of Xpert at admission point contributed to same-day TB diagnosis and treatment in about 93% of all suspected TB patients and reduced TB mortality.
- These findings suggest that 24 h of TB services using molecular TB diagnostic tools should reduce in-hospital TB delays and mortality due to TB care cascade gaps.

## Background

Despite falling tuberculosis (TB) incidence rates globally and in the WHO African Region, Mozambique shows an increasing trend of TB incidence within the last 5 years, and in 2017, it was ranked in the 11th place among the 20 countries with the highest burden of TB, with incidence rate estimated at 551/100 000 inhabitants in the general population [1].

Mozambique has a national policy on TB infection control (TBIC) in healthcare facilities, congregate settings, and households [2]. However, barriers to operationalization and adherence to TBIC measures within healthcare facilities remain [3, 4], which is contributing to TB diagnostic and treatment delays, onward TB transmission, and death [3, 5, 6].

In most of the health facility settings in Mozambique, TB care cascade gaps are common. Suspected TB patients are admitted directly to the general medical wards, frequently without an adequate screening for TB at the admission point, where they wait to be tested. Unfortunately, even after their hospital admission, many still experience delays of weeks or months before getting thoroughly examined for TB diagnosis and treatment. In addition, hospital TB mortality persists in Beira city, in part due to TB diagnosis and treatment delays related to logistical and operational barriers within a healthcare system or in-ward environmental which hinder the impact of government investments on TB infection control and prevention in the last 5 years [7–9].

The lack of designated logistic TB staff and limited TB services within the health facilities in Mozambique are known to be important factors associated with the lack of expedition of sputum collection and its examination results, and accumulation (overload) of untested sputum samples, respectively [5, 7, 8]. Because of these shortcomings, the median (IQR) time delay of Xpert and smear microscopy sample testing response from sample collection was about 10 (9) days in 2018 instead of the normally expected 2 h [7] and 62 (83) days in 2013 [5], respectively, due to health system delays, within the public health facilities in Beira city.

Appointing a cough officer nurse to deal with and expedite all TB matters is an intervention that has proven to be effective at decreasing delays in TB diagnosis and treatment in other contexts [10–12].

However, nurses are overwhelmed in Mozambique, as the country’s nurse to population ratio is about 2.9 per 10 000 inhabitants [13]. The use of trained laypersons in hospital settings in Kenya was successfully observed to be helpful on early TB case detection and treatment [14]. On the other hand, having 24-h TB laboratory services, ensuring routine and urgent TB diagnostic tools (GeneXpert MTB/RIF assay, for instance) available every day, including weekend and (inter) national holidays, is an effective intervention, not only in reducing nosocomial transmission of TB but also in speeding up early TB diagnosis and treatment [7, 12, 15].

The use of hospital auxiliary workers (HAWs) by the Mozambique Ministry of Health to support and expedite all logistic TB matters within the medical wards of public health facilities is on-going as a routine task of this category of workers. However, published data on the contribution of this approach to the reduction of TB diagnosis and treatment delays and implementation of infection prevention and control measures in healthcare facilities is unavailable. On the other hand, despite clinical laboratories of the public hospitals are open 24 h per day, it is quite common that TB laboratories are closed after 3:00 PM, on weekends, or on (inter) national holidays [7]. In addition, little is being done around the country in relation to the availability of 24-h TB laboratory services as one of the main strategies for TB infection prevention and control [2, 16].

To address these logistic management barriers in relation to TB diagnosis and treatment delays, an implementation science study was conducted. The aim was to assess whether the use of HAWs and the availability of 24-h TB laboratory services using Xpert should reduce delays in TB diagnosis, treatment, and hospital TB mortality. The study was carried out at Beira and Nampula Central Hospitals, as intervention and control sites, respectively.

## Material and methods

### Study design

Quasi-experimental studies [17] were used to assess the contribution of HAW and 24-h TB laboratory services on TB diagnosis, treatment delays, and TB mortality at HCB. Data were collected retrospectively from medical records for the 6-month period (from September 2017 to February 2018) preceding implementation in both arms (intervention and control sites) and compared to the 6-month period (from September 2018 to February 2019) post-implementation.

### Study settings

The study was conducted in Beira Central Hospital (HCB) as an intervention site and Nampula Central Hospital (HCN) as a non-equivalent control site, chosen for convenience. Beira city (the capital of Sofala Province) is located in the central region of Mozambique and is the second largest city in Mozambique, after Maputo city, the capital of Mozambique. In 2019, the city of Beira had about 465 918 inhabitants of which 50% were male [18].

HCB is a referral hospital for three provinces (Sofala, Manica, and Tete) and partly for the northern region of Inhambane Province. Most of the TB-suspected patients from these provinces are referred to this TB laboratory for diagnosis, as it is the only one in the region equipped with high-skilled personal and TB laboratory technology, including GeneXpert MTB/RIF, culture, drug sensitivity test (DST), and line probe assay (LPA) for *Mycobacterium tuberculosis* complex. The hospital has seven medical wards with about 300 beds, including two wards and 40 beds for TB patients [19].

HCN is located in the northern region of the country, and it is also a referral hospital for three provinces (Nampula, Cabo-Delgado, and Niassa) and partly for the northern districts of Zambézia Province. In the HCN, almost all services and equipment are the same as those existing in the HCB. The HCN is the only facility in the northern region performing MTB culture, LPA, and DST; hence, most of the TB-suspected patients are referred to and diagnosed. The flow chart of suspected TB patients is entirely the same as HCB. HCN has four medical wards with a capacity of about 250 beds, including two wards and 50 beds reserved for TB patients. Every suspected TB patient from the admission room is sent directly to medical wards together with other kinds of patients (there is no isolation room) until the TB diagnosis is made. Those with laboratory-confirmed TB are transferred out to the TB treatment wards. Importantly, weekend sputum examination is not performed, and inward delays in TB diagnosis and treatment are common [20].

### Population, sampling, sample size, and exclusion criteria

Using a quasi-experimental design, probability sampling is not applicable as the intervention aims to measure a new hospital-wide model for care which will be implemented throughout the hospital [17]. Therefore, all suspected pulmonary TB patients with hospital admission were eligible. Having HAW as an expediter of logistic TB matters and 24-h TB laboratory services for all suspected pulmonary TB patients during their hospitalization was considered as the primary point, and early TB diagnosis, treatment, and TB mortality as the endpoint. However, suspected TB patients with psychiatry disorders or with unconsciousness, younger than 18 years old, patients with confirmed TB, and in treatment or with extrapulmonary TB were excluded from the study.

### Study implementation strategy, intervention, and intervention logic pathway

#### Implementation strategy

Ten HAWs were chosen for convenience and received 5-day training on (a) biosafety and sputum collection, (b) logistics matters for early TB diagnosis and treatment, and (c) evidence-based TB patient education curriculum and psychosocial support. Similarly, all TB laboratory technicians participated in 3-day training on biosafety and sputum examination using national protocols for sputum smear microscopy and GeneXpert MTB/RIF examination procedures [21, 22]. The trainings were conducted by local healthcare professionals in collaboration with the research team.

The objective of the implementation strategy was to ensure that HAWs have knowledge of and are implementing the terms of reference of a hospital-based expediter of logistic TB matters (as described in Table 1), and TB laboratory technicians are engaged to the early TB diagnosis and implementation of hospital TB control infection and prevention measures.

**Table 1:** Terms of reference of the hospital-based expediter of logistic TB matters—Beira, Mozambique

In collaboration with medical doctors, to identify TB suspected patients in the emergency room or in the medical wards daily (24 h per day) and ensure that sputum request form is correctly filled out, sputum sample is immediately collected at admission point and sent to TB laboratory as soon as possible. If the result of sputum examination through “on-the-spot strategy” is negative, another sputum sample must be collected in the next morning and send to the TB laboratory as soon as it is collected.	
In collaboration with laboratory technicians, collect sputum smear microscopy or Xpert MTB/RIF results from the TB laboratory, in every 3 h, and give them immediately to medical doctors in order to ensure early treatment initiation for those patients with positive MTB.	
To ensure the provision of the N95 masks and prompt isolation of all pulmonary TB patients as soon as the diagnosis is made, taking them out from emergency room/general medical ward directly to the TB ward.	
In collaboration with medical ward nurses, to ensure personalize psychosocial support and conclusion of evidence-based curriculum of TB patient education (within 3 days) to all TB patients (including their families and visitors whenever possible) about the misunderstanding of the TB, its cause, modes of transmission and prevention, treatment and related side effects, reasons for 6-month period (or more) of TB treatment, and the consequences of giving TB treatment up.	

#### Intervention

The intervention was implemented within the 6-month period (from September 2018 to February 2019). The main interventions were having HAWs (within the medical wards and at admission point) and TB laboratory technicians, as expediter of logistic management TB matters and ensuring 24-h TB laboratory services, including weekends and (inter) national holy days, respectively.

The HAWs were not devoting to exclusively attending to TB duties but also were coordinating/collaborating with emergency room and medical ward teams, and therefore, other workplaces were not being neglected. Similarly, TB laboratory technicians were integrated into 24-h work shifts/schedules to overcome the limited working hours of the TB laboratory.

Before implementing the intervention, meetings were held with all managers, medical doctors, and nurses, in collaboration with the hospital leadership aiming to inform and ask support for effective logistic management of commodities and changes on working hours of the hospital TB services and flow chart for sputum sampling collection, examination, and delivery of positive results.

In addition, the hospital leadership recommended that any HAW and/or TB laboratory technician who should integrate into 24-h work shifts/schedules and has more than 40 working hours per week should be paid stipends according to the Mozambique Ministry of Health regulations for extra working hours.

#### Intervention logic pathway

The intervention package was given to all eligible patients; however, data collection and documentation were done only for those who signed the written informed consent. The package was composed of (a) sputum collection at admission point, (b) expedition of TB diagnosis and treatment initiation, and (c) TB patient education and psychosocial support during the hospitalization. Details of each component are given below.

##### Immediate collection of sputum sample

In collaboration with medical doctors, the HAW equipped with the respiratory N95 masks were responsible to identify suspected pulmonary TB patients with hospital admission but still in the emergency room (admission point) or in the medical wards and ensure that sputum request form is correctly filled out and the sputum sample is immediately collected at any time—“on-the-spot strategy”—and send to TB laboratory as soon as possible. If the result of sputum examination through “on-the-spot strategy” was negative, the second sputum sample should be collected the next morning and send to the TB laboratory as soon as it is collected.

For the biosafety purposes, patients were guided to step off the emergency room or general medical wards to produce a sputum sample in the open air (previously identified) and away from other people to avoid possible transmission of *M. tuberculosis* or other airborne pathogens. HAWs were instructed to help the patients to fasten the lid (or to do it if the patient is unable) once the sputum sample has been added. Then, the HAW could safely transfer the sputum sample to the staff on the TB laboratory.

##### Expedition of TB diagnosis and treatment initiation

To overcome the limited TB laboratory services provision, which seems to contribute to sputum sample overload and, consequently, delays in TB diagnosis and treatment initiation, work shift or scheduled work of TB laboratory technicians were designed according to the Ministry of Health (MoH) regulations.

Having 24 h of TB laboratory technicians, the sputum sample collected by the HAW at the admission point or general medical wards and sent to the TB laboratory was examined as soon as the technician received it.

In collaboration with the laboratory technicians, the HAWs were instructed to collect, in every 3 h at maximum, all sputum examination results, from the TB laboratory to the medical doctor’s office.

As soon as the diagnosis of pulmonary TB was made, the HAWs were isolating the TB patients by taking them to the TB wards, initiating TB treatment and ensure the provision of the respiratory N95 masks to healthcare workers, patients, and visitors.

##### TB patient education and psychosocial support

An evidence-based TB patient education curriculum was designed, adopted from the Centers for Disease Control and Prevention (CDC) TB patient education material [23]. There were four main topics designed to explain key points of local misunderstanding about (a) facts of TB infection and disease; (b) modes of transmission and protecting family and friends; (c) facts of TB treatment and related side effects, including reasons for the 6-month period (or more) and the consequences of giving TB treatment up; and (d) connection between TB and HIV and its effective control measures.

In collaboration with medical ward nurses, HAWs were ensuring two kinds of TB education sessions: individual and in groups. The individual sessions, also known as personalized psychosocial support, were given in the treatment room, once per day; while the sessions in groups were given twice per day, during lunch and dinner times.

The topics could be repeated as many times as necessary to ensure further understanding of TB facts in relation to the local beliefs (like TB is a matter of witchcraft, TB has no cure, everyone who has TB also has HIV, using the same tableware with TB patients may have tuberculosis). In addition, the TB education sessions were also directed to TB patient family members and friends during their hospital visits, whenever possible.

### Data collection, variables, and implementation outcome

Data were extracted from the laboratory and treatment registration books, including demographic and clinical data such as sex, age, HIV and treatment status, WHO clinical stages of HIV, sputum smear microscopy result, and GeneXpert sputum results, as well as (a) the date recorded for hospital admission, (b) the date recorded for positive smear microscopy and/or GeneXpert assay, (c) the date recorded for anti-TB treatment initiation, and (d) the medical outcome of the TB patients admitted to medical wards.

The main study implementation outcomes were (a) time from hospital admission to sputum examination results, (b) time from hospital admission to treatment initiation, and (c) proportion of same-day TB cases diagnosed, initiated TB treatment, and TB patient with unfavorable outcome after hospitalization (hospital TB mortality).

### Data analysis

Descriptive statistics (frequencies, percentages, medians with interquartile range (IQR)) were used to summarize patient characteristics. Time delays from admission to TB diagnosis and treatment initiation and TB mortality after hospital admission were assessed. Differences in the proportions across the groups were compared using the chi-square test of proportions. Mann-Whitney *U* nonparametric test and logistic regression and its adjusted odds ratio (aOR) at 95% confidence interval were used to assess the difference and the effect of intervention, respectively, of the median time delays from hospital admission to TB diagnosis, TB treatment initiation and hospital TB mortality between the two groups (intervention and control sites) in both, before and after intervention periods, respectively. We consider a significance level of 5% for all statistical analysis. Statistical Package for Social Sciences (SPSS) version 20 for Windows was used for data analysis.

## Results

### Clinic-epidemiological characteristics of the TB patients

A total of 522 TB patients were included in the analysis, of which 52% are from the intervention site. The period before the intervention, clinic-epidemiological data of 219 TB patients from both sites were retrieved and 103 (47%) from the intervention site. While in the intervention phase, 303 TB patients were enrolled and followed up, of which 167 (55%) were beneficiaries of the interventions and 136 (46%) were in the control site.

From the total, 302 (58%) were males and 220 (42%) females, median (IQR) age was 34 (16) years and the most frequent (34%) age category was 25–34. About 367 (72%) patients were TB/HIV co-infected, and only 162 (43%) were on HIV treatment at admission (Table 2). In general, the clinic-epidemiological characteristics (sex, age, and history of TB disease) were similar (*P* > 0.05) in both sites, either before or after the intervention, except for HIV and antiretroviral treatment status that had statistically differences (*P* < 0.05).

**Table 2:** Clinic-epidemiological characteristics of the TB patients admitted to Beira and Nampula Central Hospitals, Mozambique

Characteristics	Before intervention			After intervention		
	Control site, no.	Intervention site, no.	*P* value (chi-square)	Control site, no.	Intervention site, no.	*P* value (chi-square)
Sex						
Male	67	59	0.077	77	99	0.658
Female	49	44		59	68	
Total	116	103		136	167	
Age categories						
15–24 years old	23	12	0.099	23	32	0.147
25–34 years old	39	44		43	51	
35–44 years old	29	23		32	41	
45–54 years old	13	16		17	27	
> 55 years old	12	8		21	16	
HIV status						
Positive	72	84	0.010	72	139	0.010
Negative	44	19		64	28	
Highly active antiretroviral treatment (HAART)						
On HAART	11	37	0.001	41	73	0.001
Not on HAART	105	66		95	94	
History of TB treatment and sensitivity to drugs						
New case	77	66	0.106	78	91	0.106
Relapse	33	25		43	45	
MDR TB	6	12		15	31	

### Fidelity to deliver the intervention and implementation strategy

The delivery of the intervention through the expected logic pathway was measured either by intervention delivery forms or by questionnaire applied to patients. About 97.9% and 81.6% of all patients with 1 week or more of hospitalization confirmed to receive, at least, two times and three times, respectively, all expected deliverables. The extra hours’ stipend, the regular provision of N95 respiratory masks, supervision, and effective communication were the main motivational influences by the TB laboratory technicians and HAWs.

The implementation strategy was delivered as planned among almost all the main stakeholders (TB laboratory technicians, medical doctors, nurses, and HAWs, as well as visitors). The trainings on biosafety and Xpert examination procedures, the regular provision of N95 respiratory masks, and the engagement of the program managers and hospital decision-makers seem to have contributed successfully to the fidelity to deliver the implementation strategy. Supervision and effective communication played an important role in the successful implementation of the intervention.

### Time from hospital admission to laboratory TB diagnosis

**Fig. 1:**
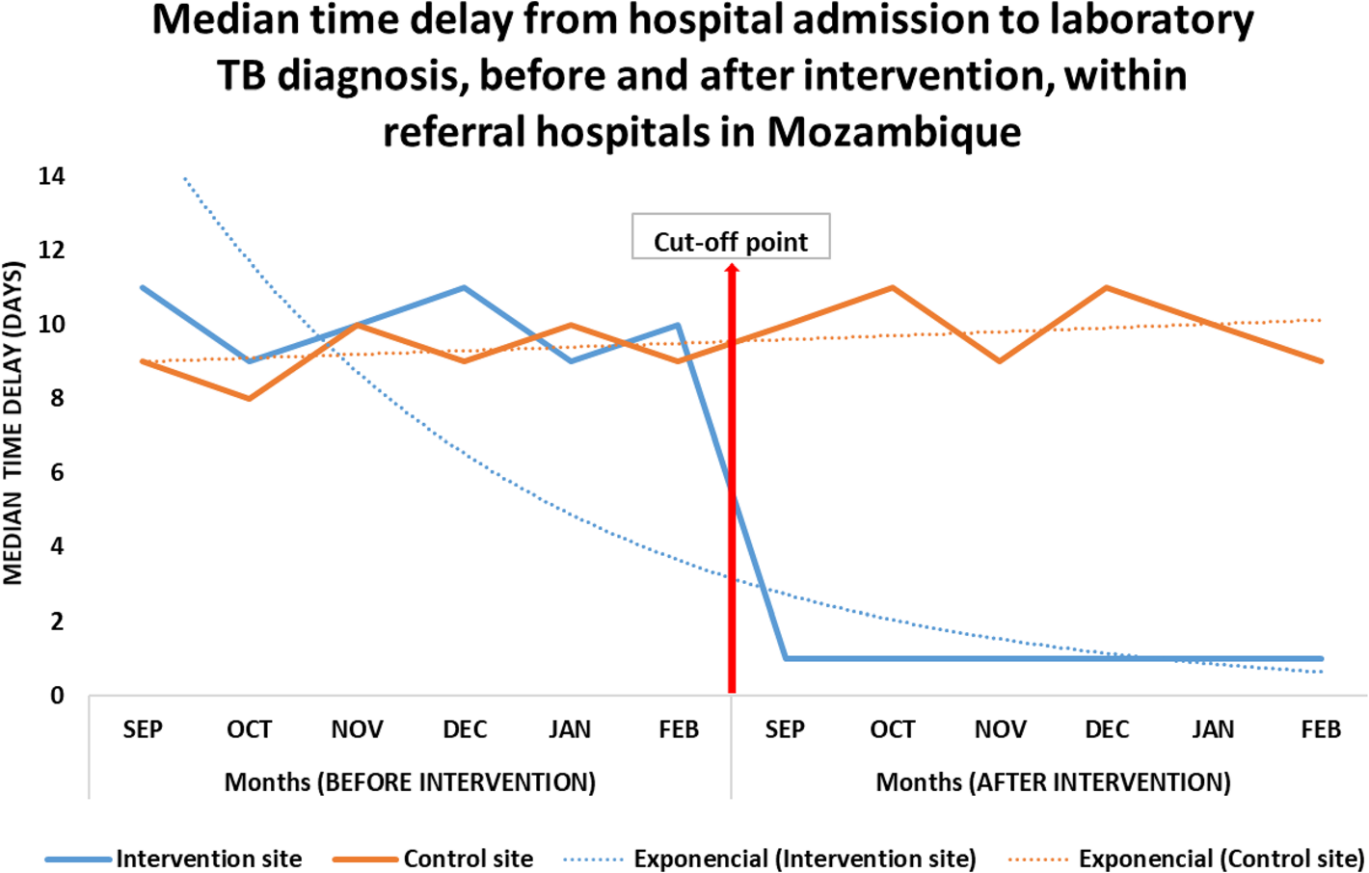
Median time delay from hospital admission to laboratory TB diagnosis, Beira and Nampula Central Hospitals, Mozambique (produced by authors)

The total median (IQR) time delays from hospital admission to TB diagnosis before the intervention in both control [9 (3) days] and intervention [10 (2) days] groups were statistically similar (*P =* 0.199). However, after the implementation of the intervention, there were statistically significant differences (*P =* 0.001) in the median (IQR) time delays in TB diagnosis within the control group [10 (3) days] in comparison with the intervention group [1 (1) day] (Fig. 1).

### Time from hospital admission to TB treatment initiation

**Fig. 2:**
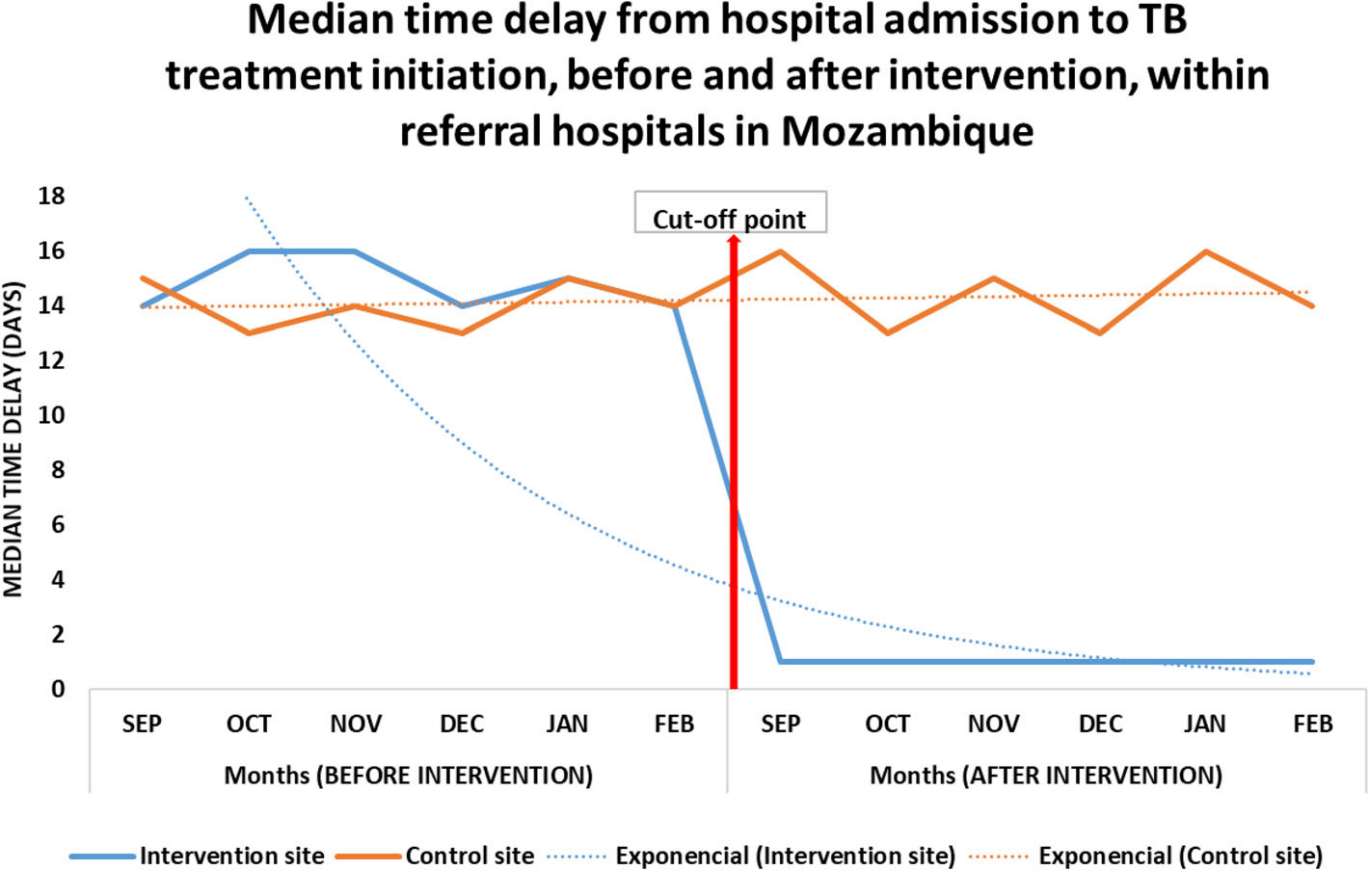
Median time delay from hospital admission to TB treatment initiation, Beira and Nampula Central Hospitals, Mozambique (produced by authors)

In the intervention site, in terms of delays from hospital admission to TB treatment initiation, a statistically significant difference (*P =* 0.001), from a median (IQR) time of 15 (3) days before the intervention to a median (IQR) time of 1 (1) day after intervention, was observed. In addition, the data indicated there was no significant difference in the control group (*P =* 0.191), between the medians (IQR) and time delay of 14 (4) days and 15 (2) days observed before and after the intervention period, respectively (Fig. 2).

### Proportion of same-day TB patients diagnosed, initiated TB treatment, and TB mortality

Every suspected TB patient admitted to medical wards was sent to HAW for TB matter expedition (early diagnosis and treatment initiation) together with 24-h TB laboratory performance, TB education, and follow-up during the hospitalization period.

The HAWs, working as logistic TB matter expediter, had contributed for early sputum sample collection and same-day TB diagnosis of about 84% (141/167) of all suspected TB patients at admission point, through “on-the-spot strategy,” and about 93% (156/167) of all TB patients have started TB treatment initiation within 24 h, in comparison with 7% of same-day TB diagnosis and 6% of treatment initiation observed in the control hospital. In addition, immediate isolation of these 156 TB patients was ensured. In terms of hospital TB mortality, the data show that after the intervention period, there is a statistically significant difference (*P* = 0.001), with 12.6% of TB mortality in the intervention hospital against 49.3% in the control hospital (Table 3).

**Table 3:** Intervention and hospital outcomes in Beira and Nampula Central Hospitals, Mozambique

Outcome measures	Before intervention					Before intervention				
	Control site	%	Intervention site	%	*P* value	Control site	%	Intervention site	%	*P* value
Sputum sample collection site										
Admission point	7	6.0	9	8.7	0.395	12	8.8	141	84.4	0.001
Medical ward	109	94.0	94	91.3		124	91.2	26	15.6	
TB diagnosis after hospital admission										
Median time of 1 day	6	5.2	7	6.8	0.517	9	6.6	141	84.4	0.001
Median time of 10 days	110	94.8	96	93.2		127	93.4	26	15.6	
TB treatment initiation after hospital admission										
Median time of 1 day	6	5.2	7	6.8	0.901	8	5.9	156	93.4	0.001
Median time of 14 days	110	94.8	96	93.2		128	94.1	11	6.6	
Health outcome among TB patients										
Hospital discharge	62	53.4	47	45.6	0.093	69	50.7	146	87.4	0.001
Hospital deaths	54	46.6	56	54.4		67	49.3	21	12.6	

### Implementation outcomes and its correlates

In terms of the effect of intervention, the data analysis showed that suspected TB patients admitted to the intervention hospital were nine times more likely to obtain an early laboratory diagnosis of TB (aOR = 9.21, 95% CI 7.52–12.11), when compared with those who were admitted to the control hospital, adjusting for other factors.

Similarly, it seems that the intervention has an impact of six times (aOR = 6.03, 95% CI 4.71–8.93) more likely to reduce delays in TB treatment initiation from hospital admission, when compared to those patients who were admitted to the control hospital, adjusting for other factors.

Unfortunately, TB patients admitted to the control hospital have eight times (aOR = 8.31, 95% CI 5.93–10.71) more likely to die when compared to patients in the intervention hospital, adjusting for other factors. This result suggests that the intervention has a possibility to avert the hospital TB mortality when compared to pre-intervention results (aOR = 1.29, 95% CI 0.83–2.42), adjusting for other factors (Table 4).

**Table 4:** Implementation outcomes and its correlates in Beira and Nampula Central Hospitals, Mozambique

Outcome measures	Before intervention		*P* value	After intervention		*P* value
	Adjusted odds ratio (aOR)	95% CI (min-max)		Adjusted odds ratio (aOR)	95% CI (min-max)	
Sputum sample collection site [reference, control hospital]						
Admission point	**0.96**	0.01–2.07	0.811	**10.97**	9.48–11.79	0.001
Lab TB diagnosis [reference, control hospital]						
Median time of 1 day	**0.83**	0.05–1.90	0.628	**9.21**	7.52–12.11	0.001
TB treatment initiation [reference, control hospital]						
Median time of 1 day	**1.01**	0.61–2.99	0.553	**6.03**	4.71–8.93	0.001
Health outcome [reference, intervention hospital]						
Hospital deaths	**1.29**	0.83–2.42	0.779	**8.31**	5.93–10.71	0.001

## Discussion

The study results demonstrate higher delays in TB diagnosis and treatment within the control hospital settings in comparison with the results from intervention hospital, particularly after the implementation of the intervention. This is due to the frailties of the national TB program to ensure adequate quality of TB services delivery, as suspected TB patients within hospital settings often are neither identified nor thoroughly evaluated in a timely manner. Even if TB diagnosis is confirmed, TB patients are not initiated on treatment or psychosocially supported to adhere and complete TB treatment for effective cure.

This finding is in agreement with several studies, including from Mozambique [5, 7], Tanzania [24] South Africa [25], and Pakistan [26]. Despite scaling-up of rapid molecular TB test, evidences are supporting that health system delays in TB diagnosis and treatment are due, in part, to TB laboratory operational delays (limited working hours of TB laboratories vs underutilization of molecular diagnostic tools) [7, 24, 27] and TB care cascade gaps, worsened by the lack of trained TB frontline staff to ensure expedition of logistic TB matters and linkage to and retention in TB treatment [5, 25–28].

Having trained HAW as expediter of logistic management TB issues and availability of 24-h TB laboratory services using Xpert contributed to same-day TB diagnosis and treatment. To our best knowledge, this is the first time in Mozambique to document the collection of sputum sample at admission point through “on-the-spot strategy,” which is sent immediately to the TB laboratory, and availability of 24-h Xpert TB testing has contributed efficiently to ensure that 93% of all suspected pulmonary TB patients had their TB diagnosis and treatment initiation within 1 day. Additionally, the research team noticed that Xpert examination of a single spot sputum sample “on-the-spot strategy” at admission point has a greater yield (93%) and minimizes the workload of TB laboratory technicians and TB delays, when compared to the morning sputum sample collection strategy.

This finding is consistent with those documented by Barrera et al. [29] and Shete et al. [30] in relation to same-day TB diagnosis, treatment, and significant reduction of delays. These studies are supporting the importance of expedition strategies for early TB diagnosis and treatment initiation through designating and training hospital TB staff at the frontline to ensure all logistic TB matters and using Xpert to speed up time to diagnosis and effective treatment.

Finding TB cases actively by rapid molecular test, separating safely and treating effectively (FAST) is a strategy known to address some TB implementation gaps [15, 29, 31], and it is in-line with one of the five priorities recommended by the Lancet Commission on building a TB-free world [28]. However, in resource-limited settings, TB laboratories using Xpert TB testing should perform 24 h per day to avoid either underutilization of available resources or overload of sputum sample to be examined coming from peripheral health centers [7, 12], as replacing all smear microscopy to Xpert at peripheral level may still take time in Mozambique.

One of the key innovative driving results of the intervention was the significant reduction of hospital TB mortality when compared to the control hospital, before and after the implementation phase. The TB mortality within the control hospital was unacceptable considering that TB is a bacterial infection, curable for which there are effective diagnostic tools and medicines. Despite this not being just a problem for Mozambique, as about 56% of all TB deaths amenable to healthcare are due to poor-quality TB service delivery [32, 33], the country national TB program is called to act accordingly toward TB elimination.

During the study, we noticed that the same-day diagnosis and early treatment initiation, together with the individual and collective patient education and psychosocial support, may have contributed for the reduction of hospital TB mortality, as misunderstanding of TB treatment issues, particularly for treatment adherence and side effects, was carefully addressed. This result is in line with the evidence supporting that delays in TB diagnosis and treatment initiation are associated with the development of severe forms of TB disease [34, 35] and TB mortality [7, 35–37]; therefore, prompt detection and proper anti-TB drug initiation are the key actions to avert morbimortality due to TB disease. In addition, promotion of task shifting and expansion of molecular diagnostic tools together with patient-centered high-quality TB services delivery are known as important tools to ensure effective TB infection prevention and control toward TB elimination [28, 32, 33, 38–40].

We anticipate that this study has limitations: using a quasi-experimental design, probability sampling is not applicable. Therefore, despite the availability of a similar unit and comparable data, selection threat remains as two units or institutions are rarely identical. However, a time-series non-equivalent control group design is the most powerful among all quasi-experimental alternatives design.

## Conclusion

In-hospital TB care cascade gaps and its related delays in TB diagnosis and treatment and high TB mortality in Mozambique are due to logistic and operational barriers amenable to poor-quality TB care. From our experience, we noticed that training HAWs on high-quality and patient-centered TB services delivery and assigning them to specific activities, together with 24 h of TB laboratory services using Xpert, may reduce cascade gaps and ultimately; ensure timely TB detection and proper treatment; and reduce its related mortality. Therefore, it is time to have in Mozambique a health facility-based TB quality improvement team and promote the “on-the-spot strategy” and 24-h availability of TB laboratory services using molecular diagnostic tools, particularly at peripheral health facilities, if TB is to be eliminated.

## Data Availability

The data that support the findings of this study are available from Mozambique Ministry of Health, but restrictions apply to the availability of these data, which were used under license for the current study, and so are not publicly available. Data are however available from the authors upon reasonable request and with permission from the Mozambique Ministry of Health.

## Abbreviations

aOR: Adjusted odds ratio
CIOB: Centro de Investigação Operacional da Beira
FCG: Calouste Gulbenkian Foundation
HAW: Hospital auxiliary worker
HIV: Human immunodeficiency virus
IF: Inês Fronteira
IHMT: Instituto de Higiene e Medicina Tropical
INS: Instituto Nacional de Saúde
IQR: Interquartile range
LMB: Logistic management barriers
ML: Miguelhete Lisboa
MROM: Maria do Rosário O. Martins
NTP: National TB Program (NTP)
PHM: Paul H. Mason
TB: Tuberculosis
TB-dt: TB diagnosis and treatment initiation
TBIC: TB infection control
UNL: Universidade Nova de Lisboa
WHO: World Health Organization

## References

[1] WHO| World Health Organization, editor. Global tuberculosis report 2018. Geneva; 2018. [cited 2019 Apr 14]. Available from: http://www.who.int/tb/publications/global_report/en/.

[2] MISAU (2010). Ministério da Saúde. Mozambique Policy and Plan on TB Infection Control in Health-Care Facilities and Congregate Settings.

[3] Brouwer M, Coelho E, Cd M, Brondi L, Winterton L, van Leth F. Healthcare workers’ challenges in the implementation of tuberculosis infection prevention and control measures in Mozambique. PLoS ONE. 2014;9(12) [cited 2019 Aug 6];Available from: http://www.ncbi.nlm.nih.gov/pmc/articles/PMC4266607/. 10.1371/journal.pone.0114364 PMC4266607 25501847

[4] Naidoo S, Seevnarain K, Nordstrom DL (2012). Tuberculosis infection control in primary health clinics in eThekwini, KwaZulu-Natal, South Africa. Int J Tuberc Lung Dis.

[5] Saifodine A, Gudo PS, Sidat M, Black J (2013). Patient and health system delay among patients with pulmonary tuberculosis in Beira city, Mozambique. BMC Public Health.

[6] Brouwer M, Coelho E, das Dores Mosse C, van Leth F (2015). Implementation of tuberculosis infection prevention and control in Mozambican health care facilities. Int J Tuberc Lung Dis.

[7] Lisboa M, Fronteira I, Colove E, Nhamonga M, Maria do Rosário OM. Time delay and associated mortality from negative smear to positive Xpert MTB/RIF test among TB/HIV patients: a retrospective study. BMC Infect Dis. 2019;19(18) [cited 2019 Apr 15]. Available from: https://bmcinfectdis.biomedcentral.com/articles/10.1186/s12879-018-3656-x. 10.1186/s12879-018-3656-x PMC6322291 30616533

[8] Orlando S, Triulzi I, Ciccacci F, Palla I, Palombi L, Marazzi MC (2018). Delayed diagnosis and treatment of tuberculosis in HIV+ patients in Mozambique: a cost-effectiveness analysis of screening protocols based on four symptom screening, smear microscopy, urine LAM test and Xpert MTB/RIF. PLoS One.

[9] García-Basteiro AL, DiNardo A, Saavedra B, Silva DR, Palmero D, Gegia M (2018). Point of care diagnostics for tuberculosis. Pulmonology..

[10] Shenoi SV, Brooks RP, Catterick K, Moll AP, Friedland GH (2013). ‘Cough officer’ nurses in a general medical clinic successfully detect drug-susceptible and -resistant tuberculosis. Public Health Action.

[11] Lin C-H, Tsai C-H, Liu C-E, Huang M-L, Chang S-C, Wen J-H (2010). “Cough officer screening” improves detection of pulmonary tuberculosis in hospital in-patients. BMC Public Health.

[12] Harries AD, Maher D, Nunn P (1997). Practical and affordable measures for the protection of health care workers from tuberculosis in low-income countries. Bull World Health Organ.

[13] INE| Instituto Nacional de Estatistica. Anuário Estatístico, editor. “Statistical yearbook” 2016: INE; 2017. [cited 2019 May 14]. Available from: http://www.ine.gov.mz/estatisticas/publicacoes/anuario/nacionais/anuario-estatistico-2016.

[14] Burmen BK, Mogunde J, Malika T. The use of laypersons to support tuberculosis screening at a Kenyan referral hospital. Int J Prev Med. 2018;9 [cited 2019 Apr 15]. Available from: https://www.ncbi.nlm.nih.gov/pmc/articles/PMC5981661/. 10.4103/ijpvm.IJPVM_226_16 PMC5981661 29899882

[15] van Kampen SC, Susanto NH, Simon S, Astiti SD, Chandra R, Burhan E (2015). Effects of introducing Xpert MTB/RIF on diagnosis and treatment of drug-resistant tuberculosis patients in Indonesia: a pre-post intervention study. PLoS One.

[16] MISAU (2013). Ministério da Saúde. Plano Estratégico do Sector da Saúde (PESS) 2014-2019.

[17] Polit DF, Beck CT (2012). Nursing research: generating and assessing evidence for nursing practice.

[18] INE| Instituto Nacional de Estatistica, editor. Annual projections of the population, urban and rural districts of Sofala province 2007–2040. Maputo; 2007. [cited 2019 Apr 16] p. 89. Available from: http://www.ine.gov.mz/estatisticas/estatisticas-demograficas-e-indicadores-sociais/populacao/projeccoes-da-populacao/projeccoes-2007-2040-sofala.pdf/view.

[19] Direcção Provincial de Saúde de Sofala (DPS-Sofala) (2019). Sofala anual tuberculosis report.

[20] Direcção Provincial de Saúde de Nampula (DPS-Nampula). "Nampula Provincial Health Directorate". Nampula Anual Tuberculosis Report. Nampula, Mozambique: National Tuberculosis Control Programme - Ministério de Saúde, Moçambique; 2019 p. 43–5.

[21] MISAU - Ministério de Saúde de Moçambique (2012). Baciloscopy manual of tuberculosis.

[22] MISAU - Ministério da Saúde (2014). Directrizes para a implementação do GENEXPERT® MTB/RIF em Moçambique.

[23] CDC - The Centers for Disease Control and Prevention. Patient education materials series: pamphlets, brochures, booklets. Tuberculosis. 2018; [cited 2019 Apr 18]. Available from: https://www.cdc.gov/tb/publications/culturalmaterials.htm.

[24] Mpagama SG, Mbelele PM, Chongolo AM, Lekule IA, Lyimo JJ, Kibiki GS, et al. Gridlock from diagnosis to treatment of multidrug-resistant tuberculosis in Tanzania: low accessibility of molecular diagnostic services and lack of healthcare worker empowerment in 28 districts of 5 high burden TB regions with mixed methods evaluation. BMC Public Health. 2019;19(1) [cited 2019 Apr 21]. Available from: https://bmcpublichealth.biomedcentral.com/articles/10.1186/s12889-019-6720-6. 10.1186/s12889-019-6720-6 PMC6458695 30971228

[25] Cox H, Dickson-Hall L, Ndjeka N, van’t Hoog A, Grant A, Cobelens F, et al. Delays and loss to follow-up before treatment of drug-resistant tuberculosis following implementation of Xpert MTB/RIF in South Africa: A retrospective cohort study. PLoS Med. 2017;14(2) [cited 2019 Apr 21]. Available from: https://www.ncbi.nlm.nih.gov/pmc/articles/PMC5319645/. 10.1371/journal.pmed.1002238 PMC5319645 28222095

[26] Ali SM, Naureen F, Noor A, Fatima I, Viney K, Ishaq M, et al. Loss-to-follow-up and delay to treatment initiation in Pakistan’s national tuberculosis control programme. BMC Public Health. 2018; [cited 2019 Apr 21];18. Available from: https://www.ncbi.nlm.nih.gov/pmc/articles/PMC5845151/. 10.1186/s12889-018-5222-2 PMC5845151 29523100

[27] Jacobson KR, Theron D, Kendall EA, Franke MF, Barnard M, van Helden PD (2013). Implementation of GenoType MTBDRplus reduces time to multidrug-resistant tuberculosis therapy initiation in South Africa. Clin Infect Dis Off Publ Infect Dis Soc Am.

[28] Reid MJA, Arinaminpathy N, Bloom A, Bloom BR, Boehme C, Chaisson R (2019). Building a tuberculosis-free world: the Lancet Commission on tuberculosis. Lancet.

[29] Barrera E, Livchits V, Nardell E (2015). F-A-S-T: a refocused, intensified, administrative tuberculosis transmission control strategy. Int J Tuberc Lung Dis..

[30] Shete PB, Nalugwa T, Farr K, Ojok C, Nantale M, Howlett P (2017). Feasibility of a streamlined tuberculosis diagnosis and treatment initiation strategy. Int J Tuberc Lung Dis.

[31] Le H, Nguyen N, Tran P, Hoa N, Hung N, Moran A (2019). Process measure of FAST tuberculosis infection control demonstrates delay in likely effective treatment. Int J Tuberc Lung Dis.

[32] Kruk ME, Gage AD, Arsenault C, Jordan K, Leslie HH, Roder-DeWan S (2018). High-quality health systems in the sustainable development goals era: time for a revolution. Lancet Glob Health.

[33] Pai M, Temesgen Z (2017). Mind the gap: time to address implementation gaps in tuberculosis diagnosis and treatment. J Clin Tuberc Mycobact Dis.

[34] Virenfeldt J, Rudolf F, Camara C, Furtado A, Gomes V, Aaby P, et al. Treatment delay affects clinical severity of tuberculosis: a longitudinal cohort study. BMJ Open. 2014;4(6) [cited 2019 Apr 4]. Available from: http://www.ncbi.nlm.nih.gov/pmc/articles/PMC4067883/. 10.1136/bmjopen-2014-004818 PMC4067883 24916087

[35] Jørstad MD, Aẞmus J, Marijani M, Sviland L, Mustafa T (2018). Diagnostic delay in extrapulmonary tuberculosis and impact on patient morbidity: a study from Zanzibar. PLoS One.

[36] Gaifer ZA (2017). Risk factors for tuberculosis mortality in a tertiary care center in Oman, 2006-2016. Int J Mycobacteriology.

[37] Aljohaney AA (2018). Mortality of patients hospitalized for active tuberculosis in King Abdulaziz University Hospital, Jeddah, Saudi Arabia. Saudi Med J.

[38] Vesga JF, Hallett TB, Reid MJA, Sachdeva KS, Rao R, Khaparde S (2019). Assessing tuberculosis control priorities in high-burden settings: a modelling approach. Lancet Glob Health.

[39] Dlodlo RA, Heldal E (2019). Comprehensive care for all individuals with tuberculosis is needed now. Lancet Glob Health.

[40] Pai M, Temesgen Z (2019). Quality: the missing ingredient in TB care and control. J Clin Tuberc Mycobact Dis.

